# A virtual imaging study of microcalcification detection performance in digital breast tomosynthesis: Patients versus 3D textured phantoms

**DOI:** 10.1002/mp.17873

**Published:** 2025-05-08

**Authors:** Katrien Houbrechts, Lesley Cockmartin, Nicholas Marshall, Liesbeth Vancoillie, Stoyko Marinov, Ruben Sanchez de la Rosa, Remy Klausz, Ann‐Katherine Carton, Hilde Bosmans

**Affiliations:** ^1^ Medical Physics and Quality Assessment Department of Imaging and Pathology KU Leuven Leuven Belgium; ^2^ Department of Radiology UZ Leuven Leuven Belgium; ^3^ GE Healthcare Buc France

**Keywords:** detection, digital breast tomosynthesis, digital phantoms, virtual imaging trial

## Abstract

**Background:**

Clinical studies to evaluate the performance of new imaging devices require the collection of patient data. Virtual methods present a potential alternative in which patient‐simulating phantoms are used instead.

**Purpose:**

This work uses a virtual imaging technique to examine the extent to which human observer microcalcification detection performance in phantom backgrounds matches that in real patient backgrounds for digital breast tomosynthesis (DBT).

**Methods:**

This work used the following DBT image datasets: (1) 142 real patient images and (2) 20 real images of the physical L1 phantom, both acquired on a GEHC Senographe Pristina system; (3) 217 simulated images of the Stochastic Solid Breast Texture (SSBT) phantom and (4) 217 simulated images of the digital L1 phantom, both created with the CatSim framework. The L1 phantom is a PMMA container filled with water and PMMA spheres of varying diameters. The SSBT phantom is a computational phantom composed of glandular and adipose tissue compartments. Signal‐present images were generated by inserting simulated microcalcification clusters, containing individual calcifications with thicknesses and projected areas in the range of 165–180 µm, 195–210 µm and 225–240 µm, and 0.025–0.031 mm^2^, 0.032–0.040 mm^2^, 0.041–0.045 mm^2^ respectively, at random locations into all four background types. Three human observers performed a search/localization task on 120 signal‐present and 97 signal‐absent volumes of interest (VOIs) per background type. A jackknife alternative free‐response receiver operating characteristic (JAFROC) analysis was applied to calculate the area under the curve (AUC). The simulation procedure was first validated by testing the physical and digital L1 background AUC values for equivalence (margin = 0.1). The AUC for patient backgrounds and each phantom type (SSBT, physical L1, digital L1) was then compared. Additionally, each patient's VOI was categorized in homogeneous or heterogeneous background texture distribution by an experienced physicist, and by local volumetric breast density (VBD) at the insertion position to examine their effect on correctly detected fraction of microcalcification clusters.

**Results:**

Mean AUC for the patient images was 0.70 ± 0.04, while mean AUCs of 0.74 ± 0.04, 0.76 ± 0.03, and 0.76 ± 0.07 were found for the SSBT, physical L1 and digital L1 phantoms, respectively. The AUC for the physical and digital L1 phantoms was equivalent (*p* = 0.03), as well as for the patients and SSBT backgrounds (*p* = 0.002). The physical and digital L1 images did not have equivalent detection performance compared to patient images (*p* = 0.06 and *p* = 0.9, respectively). In patient backgrounds, the correctly detected fraction of microcalcifications clusters fell from 0.53 for the lowest density (VBD < 4.5%) to 0.40 for the highest density (VBD ≥ 15.5%). Microcalcification detection fractions were 0.52, 0.55, and 0.55 for the SSBT, physical L1 and digital L1 backgrounds, respectively.

**Conclusions:**

Detection levels were equivalent between the physical and digital versions of the L1 phantom. Detection in L1 and patient backgrounds was not equivalent, however, differences in detection performance were small, confirming the potential value of this phantom. The digital SSBT phantom was found to be equivalent to patient backgrounds for DBT studies of microcalcification cluster detection performance, for the DBT system and reconstruction algorithm used in this study.

## 1 INTRODUCTION

A wide variety of breast x‐ray imaging techniques exist, and their evaluation and optimization is the task of the device manufacturers and medical physicists. On the manufacturer side, the regulatory approval of the devices requires supporting evidence to demonstrate their effectiveness. Part of this can be achieved using technical testing methods, or using focused clinical studies. To address subtle improvements, long and resource‐intensive clinical trials may be required with challenges such as collecting sufficient and appropriate patient data that meet specific requirements regarding the diversity of breast anatomy and pathology,^1^ ethical limitations, long follow‐up times, and lack of clinical imaging data for new inventions or prototype systems.

These challenges, together with the difficulty in establishing the ground truth in patient images motivated the development of virtual imaging trials (VIT).^2^, ^3^, ^4^, ^5^, ^6^ The use of virtual platforms, where the human subject is replaced with a digital twin and the imaging system is replaced with a simulated version of the device, provides an alternative approach to evaluate and optimize existing and new imaging technologies. Significant effort has been made to develop VIT platforms to simulate digital breast tomosynthesis (DBT) images with similar characteristics as experimentally acquired images.^7^ Artificial lesions can be inserted into digital breast phantoms or clinical breast images. Consequently, there is a need for computational models of breast lesions such as microcalcifications and masses that can be embedded in these breast phantoms or clinical images to create virtual cancer cases.^8^ In addition, it is essential to also have computational phantoms that are realistic representations of the breast anatomy so that simulated results mimic what would occur in patients. Successive efforts have been made to create computational anthropomorphic phantoms.^9^, ^10^, ^11^, ^12^, ^13^ Computer‐generated phantoms and lesions can be efficiently produced in large numbers with a wide variety of configurations and properties.^6^, ^9^, ^14^

The use of physical anthropomorphic phantoms is another attempt to approximate the clinical reality. Most physical phantoms recently designed to investigate DBT systems contain some form of structured background.^15^, ^16^, ^17^, ^18^, ^19^, ^20^ There are limits to the extent to which physical phantoms currently model anthropomorphic breast characteristics,^21^ and these phantoms do not fully represent the range of breast types encountered in breast imaging. Often, only a selective set of cancer models is included. Nevertheless, a physical structured phantom with variable background and detection task has a number of applications such as a “one‐shot” comparison between DBT systems.^22^

The main challenge associated with breast phantoms, both digital and physical, is to achieve a certain level of realism such that the phantoms and lesion models can accurately predict system performance in a real patient population. The required degree of realism is still an open question, but will likely depend on the radiological task being addressed.^23^, ^24^

In this study, we investigated the extent to which the performance of human observers in detecting microcalcification targets in both patient and phantom backgrounds was equivalent. To perform a fair comparison, a hybrid simulation method was used to insert the same lesions into the different background datasets. First, the VIT platform was validated by comparing the calcification cluster detection performance of human observers in both experimentally acquired phantom images and simulated images of the digital twin of the phantom. Microcalcification cluster detection was then compared in the patient images and in the images of one physical and two digital phantoms.

## 2 MATERIALS AND METHODS

Four different image datasets were generated in this work using a hybrid simulation method.^5^, ^25^ Real background images were obtained by acquiring images of patients and the L1 phantom^26^ on a clinical DBT imaging system. Simulated background images of two digital phantoms, the SSBT phantom^27^ and a virtual version of the L1 phantom, were then generated using a virtual imaging framework. Next, a set of microcalcification clusters was inserted into these four host backgrounds.

### 2.1 X‐ray imaging system

#### 2.1.1 Physical x‐ray imaging system

The x‐ray device used in this study for the real acquisitions was a GEHC Senographe Pristina DBT mammography system. The unit has a CsI scintillator‐based x‐ray detector, with a pixel size of 0.1 mm × 0.1 mm. A linear, focused anti‐scatter grid is used in both the 2D mammography and DBT modes, with the septa parallel to the chest wall edge. Only the DBT mode was investigated in this work, where a total of nine evenly spaced projections were acquired over a 25° angle. The system uses a “step‐and‐shoot” scan movement with a typical scan time of approximately 9 s.^28^

#### 2.1.2 Virtual x‐ray imaging system

A virtual version of the GEHC Senographe Pristina device was simulated using the CatSim ray‐tracing projector,^29^ configured for mammography.^30^ Mono‐energetic x‐rays of 23 keV were used for the simulations in this work. This energy was determined from the modeled mean energy of the primary x‐ray spectrum at the exit of the breast, which varied from approximately 23.0 to 23.5 keV, for the 34 kV rhodium (Rh)/silver (Ag) spectrum and breast thicknesses of 50 and 65 mm, respectively.^31^ To obtain realistic projection images, the CatSim tool includes quantum noise, electronic noise, and the quantization steps. A modulation transfer function (MTF), measured on the GEHC Senographe Pristina device, was used to include the effect of blurring from the x‐ray detector^32^; this was applied in the Fourier domain after ray‐tracing the phantom and targets. Two calibration factors are required by CatSim so that the average signal intensity (SI) and signal‐to‐noise ratio (SNR) in the simulated DBT projections match those in the real projections acquired on the clinical system. These were measured using a 50 mm‐thick PMMA block positioned on the breast support platform of the real system. All the simulated and experimentally acquired DBT projections were reconstructed with an offline proprietary version of the GEHC iterative reconstruction algorithm that is used clinically. The reconstructed voxel size was 0.1 mm × 0.1 mm × 1 mm.

### 2.2 Signal‐absent background images

Four structured backgrounds were studied: (1) clinical patient images, (2) the Stochastic Solid Breast Texture (SSBT) digital phantom, (3) the physical L1 phantom, and (4) a digital version of the L1 phantom.

#### 2.2.1 Clinical patient images

A total of 142 projection sets of normal, healthy breasts with a compressed breast thickness between 50 and 65 mm imaged on a GEHC Senographe Pristina system were collected retrospectively. Only craniocaudal projection views were included. It was confirmed by a radiologist that there were no lesions present in these images. Each set contained the nine projection images from a DBT scan. All images were acquired with automatic optimization of parameters (AOP) in standard (STD) mode. The average tube load was 4.16 ± 1.03 mAs/projection at 34 kV Rh/Ag.

#### 2.2.2 SSBT digital phantom

**FIGURE 1:**
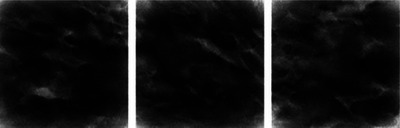
Central reconstructed DBT slice (40 mm × 40 mm) of three SSBT phantoms generated with different texture configurations.

The SSBT phantom is a computer‐generated two‐component phantom consisting of glandular and adipose tissue^27^, ^33^ (Figure 1). Three different sets of predefined texture configurations were used to generate a total of 217 phantoms of 50 mm × 50 mm with a thickness of 50 mm; each of the three configurations was represented equally. In a previous study, phantoms generated with these texture settings were rated as being visually close to patient images by radiologists.^34^ The phantoms had an isotropic voxel size of 100 µm. The volumetric breast density (VBD) of the SSBT phantoms was 12% ± 4%, calculated from the fraction of voxels identified as glandular tissue relative to the total number of voxels in the 3D voxel model. The AOP system determines the most attenuating region on a low dose pre‐exposure image, which is then used to set an exposure level. Similarly, the CatSim tool had to be calibrated to relate a certain attenuation to a tube load. A series of 50 mm thick homogeneous tissue equivalent phantoms of different glandular fractions (“CIRS Tissue Equivalent Materials” (BR0, BR30, BR70, BR100), CIRS Inc.; Norfolk, USA) were imaged on the Senographe Pristina system. Based on these measurements, a tube load of 2.97 mAs/projection was set in CatSim, and DBT projection sets of the SSBT phantoms were generated.

#### 2.2.3 L1 phantom

**FIGURE 2:**
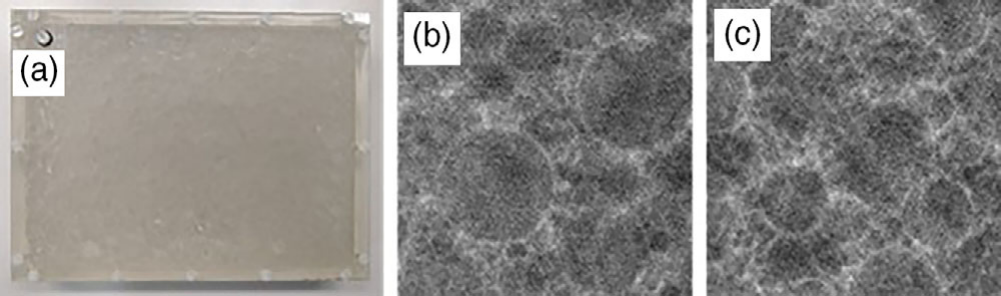
Physical L1 phantom (a), along with central reconstructed DBT slice (40 mm × 40 mm) of the physical L1 phantom (b) and the digital L1 phantom (c).

***Physical L1 phantom***: This structured phantom (Figure 2a,b) consists of a rectangular PMMA container (24 cm × 18 cm × 4.8 cm) filled with water and equal volumes of PMMA spheres of six different diameters (15.88, 12.70, 9.52, 6.35, 3.18, and 1.58 mm).^26^ The physical thickness of 48 mm approximates a breast equivalent thickness of 60 mm.^15^ Unlike the L1 phantom described by Cockmartin et al.,^15^ this version does not include lesion‐simulating objects. Twenty DBT projection sets of the rectangular L1 phantom were acquired in AOP mode, with the same system that was used for patient imaging. The average tube load was 4.32 ± 0.14 mAs/projection. The phantom was shaken before each acquisition to produce a different distribution of spheres and therefore a slightly different background in each scan.

***Digital L1 phantom***: A digital twin of the physical L1 phantom was created by filling a virtual container with exactly the same number of spheres as in the physical phantom via a sphere packing algorithm (Figure 2c). Each of the 217 phantom generations resulted in a new distribution of spheres. The digital L1 phantom is an analytical phantom, with PMMA assigned to both the container and the spheres, while the unoccupied space within the container is filled with water. The phantom edge was positioned 15 mm from the chest wall edge, and projection images were simulated with CatSim at 4.32 mAs/projection.

#### 2.2.4 Power spectrum analysis of the background images

The CatSim calibration procedure should ensure the correct relationship between signal and noise in the simulated projections. However, determining whether the simulated images with structured anatomical noise have the correct level of quantum noise, compared to physical patient and phantom acquisitions, is not straightforward. One method of examining this is to calculate the power spectrum *S(f)* in the projection images, where *f* is the spatial frequency, and compare the power spectrum magnitude over a defined spatial frequency range. The total power spectrum contains contributions from the signal power spectrum (i.e., due to structures being imaged) and from the noise power spectrum, whose magnitude at a given spatial frequency depends on the noise source, such as quantum noise, detector electronic noise, and structured noise.^35^ The contribution of quantum noise is determined by the exposure level. Cockmartin et al.^36^ showed that quantum noise starts to influence the power spectrum at progressively lower spatial frequencies as detector exposure is lowered, as is found in DBT projections compared to DM. The study by Hill et al.^37^ used a mammography system with a CsI‐based detector and found that quantum noise formed a substantial fraction of the total power magnitude at frequencies above 0.3 mm^−1^. Taking these points into consideration, the power spectrum for *f* ≳ 1 mm^−1^ was used to give an estimate of the quantum noise magnitude in the image.^36^, ^37^

The power spectrum can also be used to estimate the parameters kappa (*K*) and beta (*β*), which have been used as a means of quantifying, respectively, the magnitude and texture or correlation of image structures.^38^, ^39^, ^40^, ^41^ They are derived from a curve fit to the power spectrum calculated for the backgrounds and given in Equation (1).

(1) $$S\left(f\right)=\frac{K}{f^{\beta }}\;$$

For 100 signal‐absent images of each background, a region of 384 × 384 pixels (38.4 mm x 38.4 mm) was extracted from the 0° DICOM “For Processing” projection image. ROIs of 256 × 256 pixels were then extracted from this region in a half‐overlapping pattern. A two‐dimensional surface of polynomial degree one was fitted to each 256 × 256 ROI and then subtracted from the ROI to reduce the influence of large area trends on the power spectrum. The ROI was subsequently multiplied by a Hann window and a standard formula was used to calculate the two‐dimensional power spectrum.^42^ The radial average power spectrum $S_{avg}\left(f\right)$ was calculated excluding the 0° and 90° axes, and the parameters K and β were then calculated from a linear curve fit to the logarithm of $S_{avg}\left(f\right)$:

(2) $$log_{10}\;\left\{S_{avg}\left(f\right)\right\}=log_{10}\left(K\right)-\beta \;log_{10}\left\{S_{avg}\left(f\right)\right\}$$

In order to determine the fit range for the parameters *K* and *β*, five different spatial frequency ranges taken from the literature were applied to the power spectra of the patient images. The ranges studied were 0.07–0.45 mm^−1^, 0.15–0.70 mm^−1^, 0.06–0.81 mm^−1^, 0.20–0.70 mm^−1^ and 0.08–0.30 mm^−1^.^36^, ^37^, ^39^, ^43^, ^44^ The average coefficient of determination (*R* ^2^) was calculated for each fit range and the frequency range with the highest *R* ^2^ value was used to determine *K* and *β* for the patient and the phantom datasets.

### 2.3 Signal‐present images

#### 2.3.1 Microcalcification clusters

Three‐dimensional voxel models of suspicious microcalcification clusters, obtained in a previous study by segmenting micro‐CT images of vacuum stereotactic biopsy specimens,^25^ formed the basis of the targets in this work. Individual microcalcifications were isolated from the 3D cluster models. The size of the individual microcalcifications was determined by fitting an axis‐aligned minimum bounding box around each calcification. The thickness was defined as the largest axis measured perpendicular to the detector. The area was approximated by the area of an ellipse, with the major and minor axes defined by the bounding box dimensions parallel to the detector. Between 7 and 12 individual calcifications of equal size were then randomly selected. The calcifications were positioned in a cluster model matching the x‐, y‐, and z‐coordinates of calcifications in real clusters derived from earlier patient DBT images. Twenty unique cluster formations were used (Figure S1), with a maximum cluster volume of 11 mm × 11 mm × 5 mm. The cluster models had an isotropic voxel size of 15 µm.

Relevant microcalcification size groups were selected after a pilot study performed with 60 DBT images of each of the following backgrounds: patients, SSBT, and physical L1, where a cluster was simulated at a random location in half of the cases. The following size groups were selected to approximate an average area under the receiver operating characteristic curve (AUC‐ROC) of approximately 0.7 for each reader: three groups based on their area (XS: 0.025–0.031 mm^2^, S: 0.032–0.040 mm^2^, M: 0.041–0.045 mm^2^) and three groups based on their thickness (XS: 165–180 µm, S: 195–210 µm, M: 225–240 µm). In total, 120 microcalcification cluster models were created with 20 clusters per size group.

#### 2.3.2 Hybrid insertion method

For each of the four background types, 120 signal‐present DBT images were created by including the cluster models in the background images. First, each 3D cluster model was randomly positioned freely in air, at least 2 cm from the chest wall edge of the detector, at a random height between 10–40 mm above the table surface in the CatSim platform. DBT projection images of the cluster in air were then generated, but with quantum and electronic noise disabled during the imaging process. This resulted in a projection image that can be represented by:

(3) $$I=I_{0}e^{-\mu_{calc}\cdot t_{calc}}$$

where $I_{0}$ and I are respectively the incident and transmitted signal intensities, and *μ*_calc_ and *t*_calc_ are the linear attenuation coefficient and thickness of the calcification, respectively. To create templates, the simulated projection images of the cluster in air were divided by the simulated projection images of air only (i.e., $I_{0}$), resulting in templates represented by the ratio of $I/I_{0}$ or $e^{-\mu_{calc}\cdot t_{calc}}$. Each template consisted of a set of nine projections and contained values ranging from 0.0 to 1.0, where 1.0 represented background areas, and values less than 1.0 indicated the presence of calcifications. These templates were then multiplied into the projection image sets of the four backgrounds. At the calcification sites, the original pixel values of the background image were scaled by $e^{-\mu_{calc}\cdot t_{calc}}$. This method is a variant of the “voxel addition” method.^24^ Calcium oxalate, weighted by 0.84, was assigned as cluster material.^45^ For all backgrounds, the lesion locations were randomly chosen. After the insertion process was completed, each set of nine projections was reconstructed using an offline version of the standard clinical GEHC reconstruction algorithm.

Table 1 summarizes the modeling steps involved for the four background types, giving a basic indication of which physical processes are modeled, which are inherited from the background image, and which are not modeled. Scattered radiation is not modeled, based on the assumption that its impact on detection is small due to the presence of an anti‐scatter grid in DBT mode.

**TABLE 1:** Summary of the basic physical properties of the four image datasets.

	Image type			
Physical property	Patients	SSBT	Physical L1	Digital L1
x‐ray spectrum: background	REAL	SIM	REAL	SIM
x‐ray spectrum: lesions	SIM (23 keV mono)	SIM (23 keV mono)	SIM (23 keV mono)	SIM (23 keV mono)
Sharpness of background structures	REAL	SIM	REAL	SIM
Sharpness of lesions	SIM	SIM	SIM	SIM
x‐ray noise in background	REAL	SIM	REAL	SIM
x‐ray noise in lesions	N/M	N/M	N/M	N/M
Scattered radiation: background	REAL	N/M	REAL	N/M
Scattered radiation: lesions	N/M	N/M	N/M	N/M

*Note*: REAL indicates that the property is captured during the imaging process in real images acquired on the imaging system, SIM indicates that the process is simulated in CatSim. N/M indicates that the property is not modeled. The table does not give an exhaustive list of the properties that were not modeled.

### 2.4 Reader study

#### 2.4.1 Data preparation

From the reconstructed datasets of the four backgrounds, 120 signal‐present and 97 signal‐absent volumes of interest (VOIs) with dimensions of 40 mm × 40 mm × 40 mm were collected. For the patient images and the physical L1 phantom, the signal‐absent VOIs were extracted from random locations inside the breast and phantom boundaries, respectively. For the SSBT and digital L1 datasets, all VOIs were extracted from the DBT image with a 2 cm offset from the chest wall edge and vertically centered. The microcalcification clusters were randomly located within the signal‐present VOIs.

#### 2.4.2 Image reading

For each background, a reader study consisting of four reading sessions with a maximum of 55 cases (30 signal‐absent and 24–25 signal‐present) was conducted. Three medical physicists, who were experienced in performing reading studies, were first asked to search and localize the cluster center when a cluster was thought to be present. After localization, they were asked to rate their confidence in the presence of the cluster from 1 to 4. If the readers believed no cluster was present, they could just proceed to the next case. Before the start of each study, all readers were presented with a training set of 60 cases (30 signal‐absent and 30 signal‐present). After scoring each training case, the location of the cluster was shown in the signal‐present image, and feedback was given in the case of an incorrect answer. The ViewDEX software^46^ was used to display the DBT images one by one at 100% (1:1) resolution. The scoring was performed under diagnostic reading conditions on a calibrated 12MP monitor, and the readers were able to adjust the window/level. No time limit was imposed. The ambient light level in the room was controlled during the reading sessions, with a typical level of approximately 6 lux. During the study, one reader had to be replaced with another, well‐trained reader who had similar performance for all background types.

#### 2.4.3 Statistical analysis

For each of the four different datasets, the scores of the three readers were registered. AUC values were calculated using jackknife alternative free‐response receiver operating characteristic (JAFROC) analysis.^47^ Next, a hierarchical test procedure was employed,^48^ beginning with the primary null hypothesis (H_0_)^49^ that the difference in AUC between the physical and digital L1 phantom exceeds the equivalence margin. A significance threshold (*α*) of 0.05 was used. If the primary null hypothesis could be rejected, the secondary hypothesis was tested, namely whether the difference between AUC for the microcalcification detection task in patients and a given phantom background (SSBT, physical L1, or digital L1) falls outside the equivalence interval. Here, a Bonferroni correction was applied (*α* = 0.05/3 = 0.017). An equivalence margin of 0.1 was set for both hypotheses, with the assumption that a 10% difference in diagnostic performance with the patients as reference is considered acceptable.^50^, ^51^

In a sub‐analysis, the signal‐present cases of the patient dataset were subdivided based on background texture and local breast density. The texture was characterized by a physicist with experience in mammography through a visual analysis of the reconstructed VOIs. The images were categorized as homogeneous or heterogeneous background texture distribution. The backgrounds labeled as “homogeneous texture distribution” should not be thought of as lacking in structure; rather, the texture in these backgrounds is more uniformly distributed across the VOI than those classed as “heterogeneous texture distribution”. Following this grouping, 65% of the VOIs were classified as a homogeneous texture distribution, with the remaining 35% classed as heterogeneous texture distribution.

Local breast density was quantified in patient images with Volpara VBD software^52^ (v1.5.4.0, Volpara Health Technologies Limited, Wellington, New Zealand) applied to the 0° DICOM “For Processing” DBT projection. The VBD was measured in a 1 cm x 1 cm ROI positioned at the location of the cluster before the actual cluster was inserted. Based on the local VBD at the insertion location, the signal‐present cases were classified into 4 groups: group 1 (VBD < 4.5%), group 2 (4.5% ≤ VBD < 7.5%), group 3 (7.5% ≤ VBD < 15.5%) and group 4 (VBD ≥ 15.5%).^53^ For all three phantom backgrounds, the correctly detected fraction of the 120 signal‐present cases was calculated and compared to the correctly detected fraction in the subgroups of the patient backgrounds. It was not possible to apply Volpara to the phantom images.

## 3 RESULTS

### 3.1 Signal and noise characterization of background types

**FIGURE 3:**
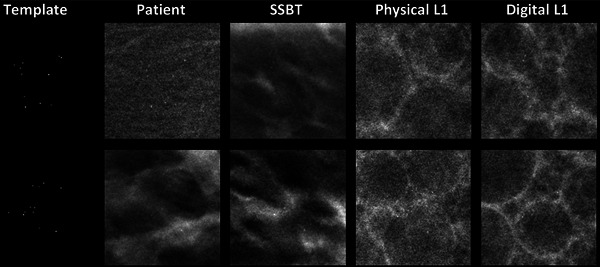
In‐plane reconstructed DBT images of two microcalcification clusters simulated in a patient image, an SSBT phantom, and a physical and digital L1 phantom.

Figure 3 presents in‐focus reconstructed DBT slices of microcalcification clusters inserted in different backgrounds, with additional examples available in Figure S2. Both the patient and SSBT backgrounds display a greater variety of texture and structures compared to the L1 backgrounds, reflecting the constraints of this phantom design.

**FIGURE 4:**
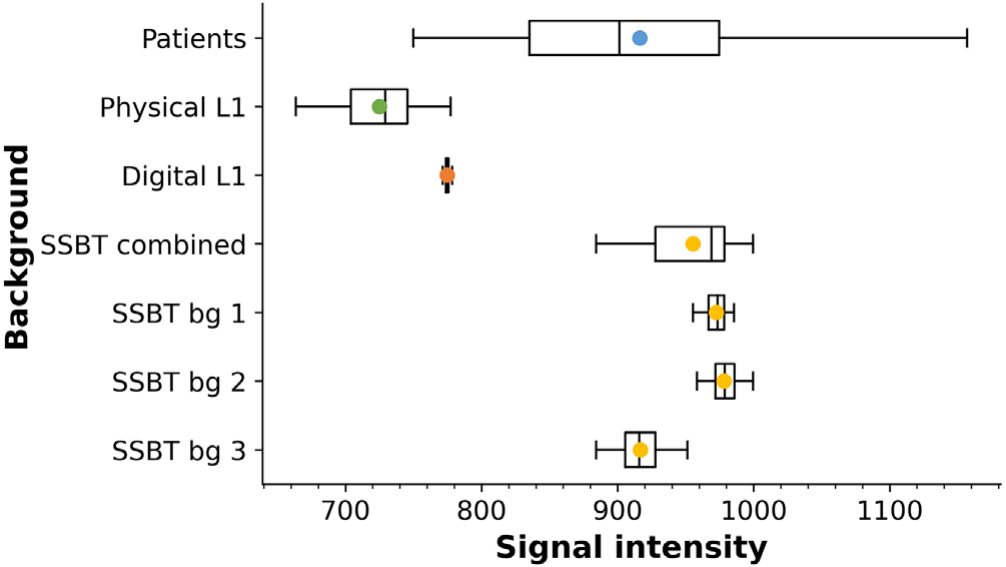
Box plot of the mean signal intensity measured in a 40 mm × 40 mm ROI in the 0° DBT projection images. The lines out from the box indicate the maximum and minimum data values, and the bottom and top of the box mark the 25th and 75th percentiles of the mean values. The line inside the box indicates the median, and the marker the mean.

Figure 4 plots the mean signal intesity (SI) averaged in a ROI of 40 mm × 40 mm measured in 100 0° central projections for each of the four signal‐absent backgrounds. Results are shown for all SSBT backgrounds combined, as well as separately for each of the three SSBT texture settings used in the study. The average SI for the SSBT backgrounds is within 4% of the patient values, while average SI results for physical and digital L1 backgrounds are respectively 21% and 16%, lower than those of the patients and SSBT images. Larger dispersion is seen in SI for the patient backgrounds compared to the phantoms.

**FIGURE 5:**
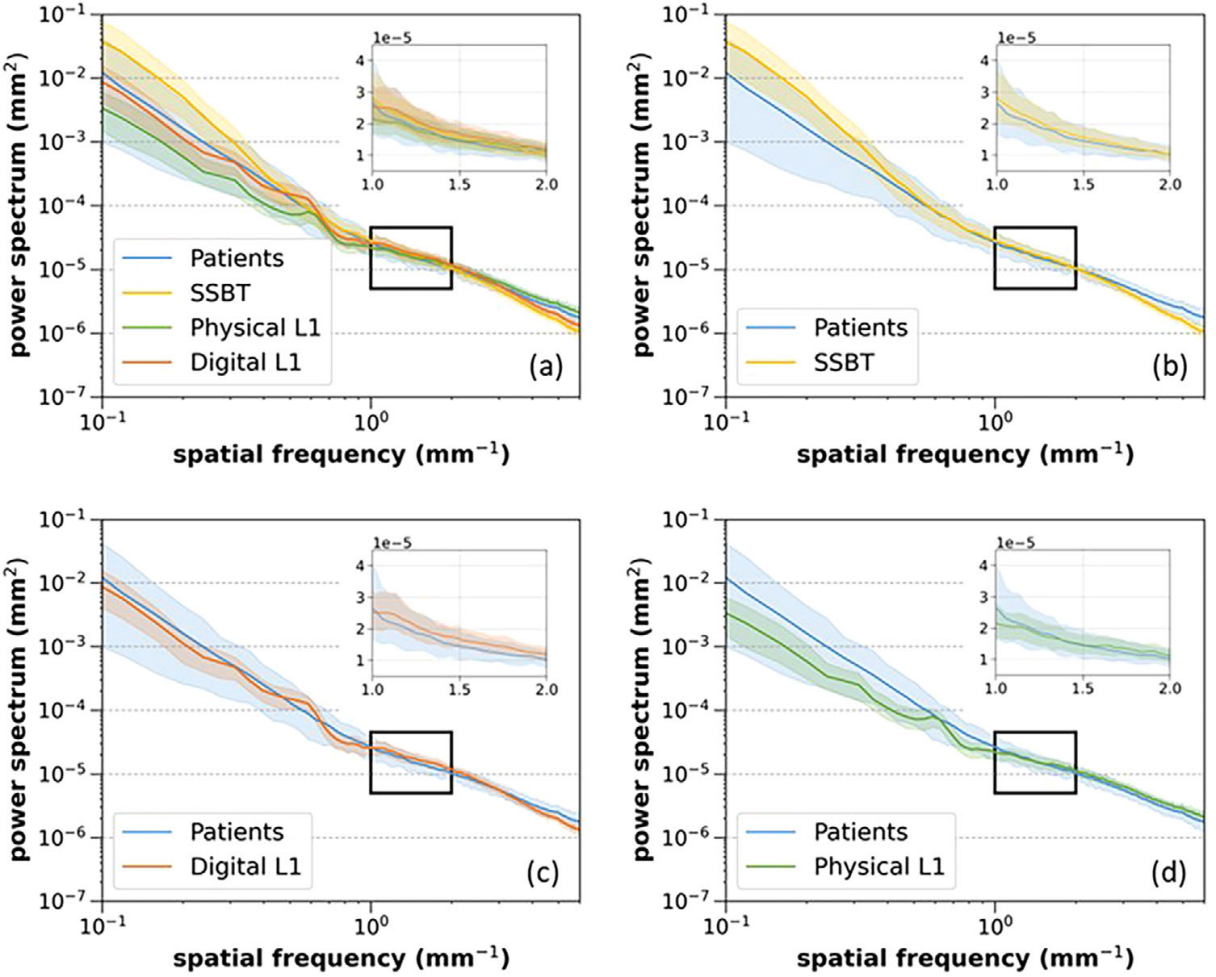
Power spectra calculated from the 0° projection from each scan, for the four backgrounds. Each solid line shows the average value for a given background, while the shaded regions show the 5% and 95% extent of the power spectra for a given background. (a) The four backgrounds compared; (b) SSBT (simulated) versus patients (real); (c) Digital L1 (simulated) versus patients (real); and (d) Physical L1 (real) versus patients (real).

**FIGURE 6:**
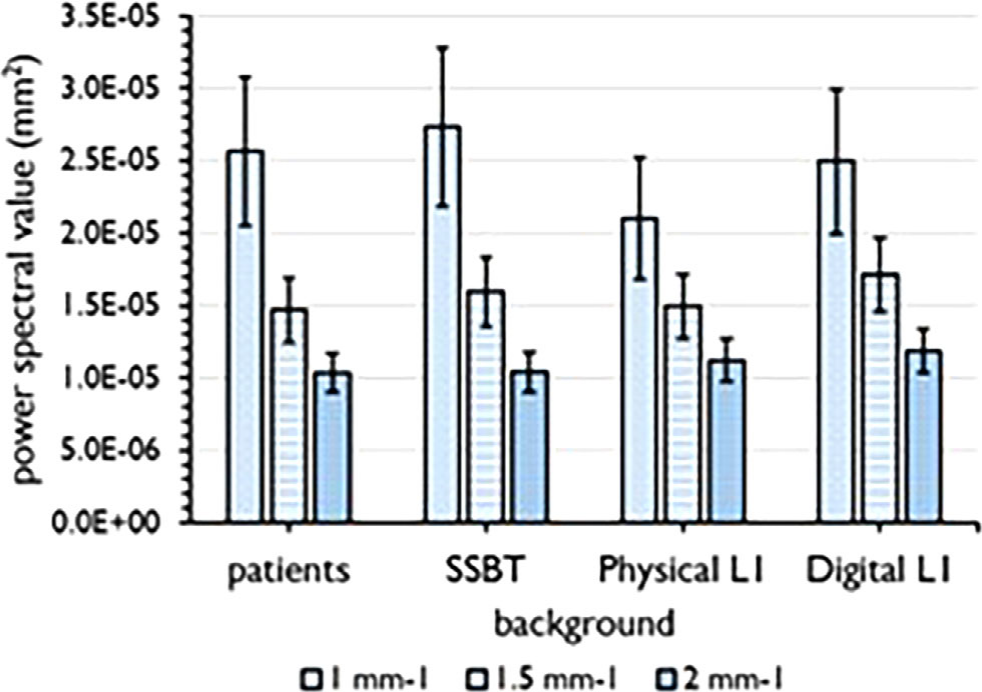
Average power spectrum value *S_avg_* at 1, 1.5, and 2 mm^−1^ calculated from the 0° projection images for each background type. The error bar indicates the coefficient of variation.

Figure 5 presents log‐log plots of the power spectra, calculated from the 0° projection images for each background type. The solid lines show the average value, while the shaded region indicates the 5% to 95% range. An inset provides a zoomed view of *S_avg_* over the region 1 to 2 mm^−1^. Figure 6 plots *S_avg_* at spatial frequencies of 1, 1.5, and 2 mm^−1^, for the four backgrounds. The error bars indicate the averaged coefficient of variation (standard deviation divided by *S_avg_*), which was approximately 20%, 15%, and 13% for the 1, 1.5, and 2 mm^−1^ data, respectively, for all background types. At 1 mm^−1^, values of *S_avg_* are similar for the patient, SSBT, and digital L1 backgrounds, while *S_avg_* for the physical L1 images is lower, although not significantly. Given the lower average SI for the physical L1 phantom images, higher quantum noise is expected for these images, but this is not seen. The insets in Figure 5 show that differences in structure noise between these backgrounds in the region of 1 mm^−1^ may explain this. At 2 mm^−1^, *S_avg_* for both the physical and digital L1 images is higher than for the patient and SSBT data and therefore more consistent with the SI values. Absolute average deviation of *S_avg_* at 1, 1.5, and 2 mm^−1^ for SSBT, L1 physical and L1 digital backgrounds was within approximately ±7% of the value for the patient images.

Table 2 lists average *K* and *β* values, along with *R* ^2^ values for the different fit ranges. Varying the fit range had minimal impact on the calculated parameters, with the most notable difference observed in the Kappa estimate when using the 0.08–0.30 mm^−1^ range. Factors influencing the frequency range for fitting include artefacts appearing at low spatial frequencies from the ROI size used in the NPS calculation and quantum noise affecting the curve fit at higher spatial frequencies.^37^ The data show the highest *R* ^2^ value of 0.97 for the fit range of 0.08–0.82 mm^−1^, which was therefore used to calculate the *K* and *β* for the patient and phantom datasets.

**TABLE 2:** Average *K* and *β* fit values for the different fit ranges, along with the *R* ^2^ values.

Study	Metheany et al. (2008)	Engstrom et al. (2009); Li et al. (2024)	Vedantham et al. (2013)	Cockmartin et al. (2013)	Hill et al. (2013)
Spatial frequency range used (mm^−1^)	0.08‐0.47	0.16‐0.71	0.08‐0.82	0.20‐71	0.08‐0.30
Kappa (± 1σ)	2.63 × 10^−5^ (± 1.88 × 10^−5^)	2.27 × 10^−5^ (± 1.01 × 10^−5^)	2.24 × 10^−5^ (± 8.63 × 10^−6^)	2.26 × 10^−5^ (± 1.11 × 10^−5^)	3.46 × 10^−5^ (± 3.93 × 10^−5^)
Beta (± 1σ)	2.48 (± 0.60)	2.49 (± 0.51)	2.49 (± 0.48)	2.51 (± 0.50)	2.49 (± 0.75)
*R* ^2^	0.95	0.95	0.97	0.95	0.93

The *K* and *β* values calculated from the curves in Figure 5 are given in Table 3. The *β* value for the physical L1 phantom is 2.22, which is 11% lower than the average of 2.49 for the pooled patient images. There is reasonable agreement between the physical and digital L1 phantom, although both *K* and *β* are higher for the digital L1 phantom. The *β* value for the SSBT background is 3.33, which is 33% higher than *β* for the patient data. Table 4 presents the *K* and *β* values for the patients when divided into subgroups based on background texture and local breast density. The *β* values increase as VBD increases, and in cases classified as heterogeneous texture.

**TABLE 3:** Parameters estimated at the lesion insertion position.

Background	Mean pixel value (± 1σ)	Kappa (± 1σ)	% difference in kappa from patient data	Beta (± 1σ)	% difference in beta from patient data
Patients (pooled)	922.3 (± 122.1)	2.24 × 10^−5^ (± 8.63 × 10^−6^)	—	2.49 (± 0.48)	—
SSBT	955.5 (± 30.5)	1.68 × 10^−5^ (± 4.13 × 10^−6^)	−25%	3.33 (± 0.22)	+34%
Physical L1	725.1 (± 27.6)	1.67 × 10^−5^ (± 2.25 × 10^−6^)	−25%	2.22 (± 0.17)	−11%
Digital L1	774.7 (± 1.6)	2.08 × 10^−5^ (± 2.64 × 10^−6^)	−7%	2.56 (± 0.16)	+3%

*Note*: Mean pixel value calculated from a 40 mm × 40 mm ROI. Kappa and Beta were measured using half‐overlapping ROIs of 25.6 mm × 25.6 mm extracted from the 38.4 mm × 38.4 mm ROI positioned at the calcification cluster insertion position, in the four background types.

**TABLE 4:** Average kappa and beta measured at microcalcification insertion position, for the homogeneous texture and heterogeneous texture patient backgrounds and for the different density groups.

Patient background	Kappa	Beta
Homogeneous texture	2.15 × 10^−5^ (± 8.34 × 10^−6^)	2.43 (± 0.50)
Heterogeneous texture	2.47 × 10^−5^ (± 8.09 × 10^−6^)	2.56 (± 0.41)
Density group 1	2.28 × 10^−5^ (± 9.06 × 10^−6^)	2.06 (± 0.38)
Density group 2	2.21 × 10^−5^ (± 6.62 × 10^−6^)	2.27 (± 0.34)
Density group 3	2.12 × 10^−5^ (± 7.42 × 10^−6^)	2.60 (± 0.41)
Density group 4	2.50 × 10^−5^ (± 9.75 × 10^−6^)	2.81 (± 0.35)

### 3.2 Calcification detection performance

**FIGURE 7:**
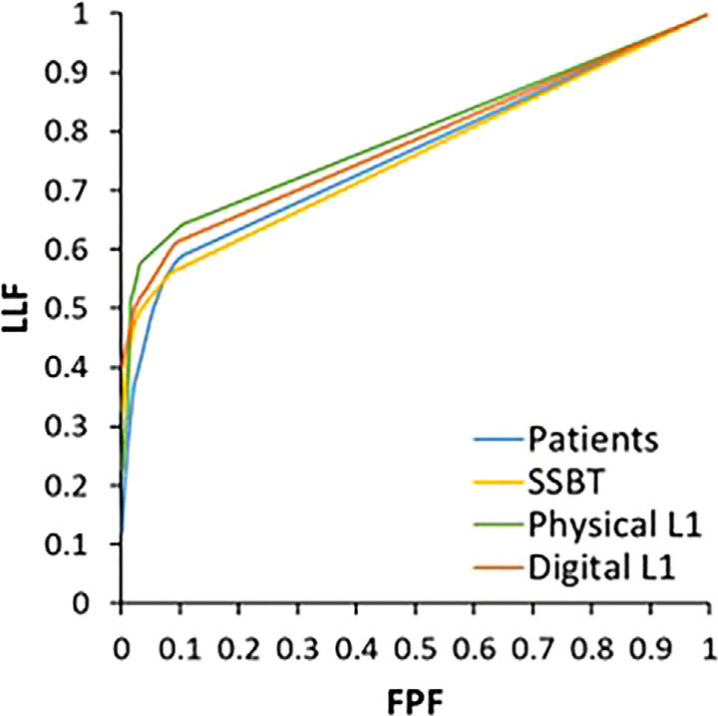
The reader‐averaged alternative FROC curves for the four different background types. FPF, false positive fraction; LLF, lesion localization fraction.

Turning to the results of the detection study, Figure 7 shows the alternative FROC curves averaged for the three readers. The curve shapes are similar for the four studied backgrounds. The mean AUC of the patient background images is 0.70 ± 0.04. For the phantoms — SSBT, physical L1 and digital L1 — the mean AUC values are 0.74 ± 0.04, 0.76 ± 0.03, and 0.76 ± 0.07, respectively. The true positive (TP), true negative (TN), false positive (FP), and false negative (FN) results of the individual readers, together with their AUC value, can be found in Table 5. The FP rate is the highest for the patient backgrounds, while the phantom backgrounds generally have lower FP rates, and therefore a higher precision. A moderate to substantial inter‐rater agreement was found between the three readers with a mean kappa of 0.53, 0.62, 0.67, and 0.64 for patients, SSBT, physical L1, and digital L1, respectively.

**TABLE 5:** The AUC of the AFROC curves for the individual readers together with the reader‐averaged AUC and the 95% confidence interval for the four backgrounds.

	Patients	SSBT	Physical L1	Digital L1
**Reader 1**				
**TP—TN—FP—FN**	59—88—22—48	65—89—15—48	71—87—22—37	63—88—15—51
**AUC**	0.72	0.74	0.77	0.73
**Reader 2**				
**TP—TN—FP—FN**	51—90—25—51	57—94—5—61	65—94—10—48	63—89—14—51
**AUC**	0.68	0.73	0.76	0.74
**Reader 3**				
**TP—TN—FP—FN**	58—87—21—51	63—95—3—56	63—96—6—52	74—95—7—41
**AUC**	0.70	0.76	0.76	0.80
**Mean AUC**	**0.70**	**0.74**	**0.76**	**0.76**
**CI 95%**	(0.66, 0.74)	(0.71, 0.78)	(0.73, 0.79)	(0.68, 0.83)
**Mean precision**	**0.71**	**0.90**	**0.85**	**0.85**

Abbreviations: FN, false negative; FP, false positive; TN, true negative; TP, true positive.

Table 6 presents the confidence intervals of AUC differences for the four backgrounds, along with the corresponding *p*‐values from equivalence testing. For the first hypothesis tested, diagnostic performance was found to be equivalent (*p* = 0.03) for the physical and digital L1 phantoms. This result supports the ability of the CatSim tool to model physical processes with sufficient accuracy relevant for microcalcification detection in digital phantom images. Regarding the second hypothesis test, the AUC in patients and SSBT phantoms is equivalent, while the difference in AUC for both the physical (*p* = 0.06) and digital (*p* = 0.9) L1 phantoms exceeded the preset equivalence margin of 0.1.

**TABLE 6:** The 95% confidence interval and *p*‐value of equivalence testing, and AUC difference for the four different backgrounds.

	95% confidence interval	*p*‐Value	∆AUC
**Physical L1 vs. digital L1**	[−0.09; 0.08]	0.03	0.004
**Patients vs. SSBT**	[0.01; 0.08]	0.002	0.04
**Patients vs. physical L1**	[0.02; 0.10]	0.06*	0.06
**Patients vs. digital L1**	[−0.02; 0.14]	0.9*	0.06

*Note*: *indicates that the calcification detection performance in both backgrounds are not equivalent (*α* = 0.05 for physical L1 vs. digital L1, *α* = 0.017 for patients vs. SSBT/physical L1/digital L1).

**FIGURE 8:**
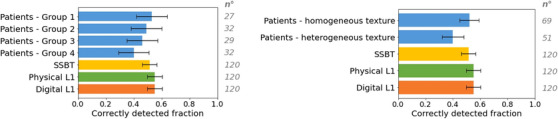
Average correctly detected fraction of the clusters simulated in the four backgrounds. The patient backgrounds are subdivided by local breast density, group 1 (VBD < 4.5%), group 2 (4.5 % ≤ VBD < 7.5%), group 3 (7.5 % ≤ VBD < 15.5%), and group 4 (VBD ≥ 15.5%) (left) and visual assessment of homogeneous versus heterogeneous background (right). The error bars indicate the 95% confidence interval.

Microcalcification detection results for the four levels of local density in the patient background are shown in Figure 8. The correctly detected fraction in patients follows the local density, falling from 0.53 for Group 1 (VBD < 4.5%) to 0.40 for Group 4 (VBD ≥ 15.5%). These can be compared with values of 0.52, 0.55, and 0.55 for the SSBT, physical L1, and digital L1 backgrounds, respectively, considering the error bars. Calcification detection in patient backgrounds classified as homogeneous texture distribution was slightly higher than in heterogeneous texture distribution backgrounds, with correctly detected fractions of 0.52 and 0.40, respectively. Calcification detection performance in the phantom backgrounds therefore corresponds most closely to that in patient backgrounds with the lowest VBD and homogeneous texture distribution.

## 4 DISCUSSION

The objective of this work was to examine whether microcalcification cluster detection performance in breast‐simulating backgrounds was equivalent to the detection performance in real patient backgrounds. If equivalent detection performance is achieved for the targets in real and simulated backgrounds, then this can be considered a form of validation of the simulated background realism^23^ and support the use of this background type in more extended studies.

As a first step, physical and digital L1 backgrounds were compared. Microcalcification detection performance was equivalent in both L1 phantom backgrounds, with an AUC of 0.76 for both cases, but with a slightly broader confidence interval for the digital L1 phantom. Calibration of CatSim ensured that the relationship between detector entrance air kerma and SI was similar for the simulations and the real system. The SI differed by less than 6% for the real and simulated L1 images, indicating that air kerma at the x‐ray detector is approximately the same. Variation in SI for the physical L1 images was higher than the variation seen for the digital L1 data, probably due to some additional variation arising from AOP short‐term reproducibility that is not present in the digital L1 images. The power spectra for the real and simulated L1 images are within 15% at 1.5 and 2 mm^−1^, which is consistent with similar levels of quantum noise in the digital and physical L1 projection images.^36^, ^54^ Above 3 mm^−1^, the power spectrum is lower for digital L1, likely due to some aspects of detector performance not being modeled in the CatSim tool.^32^

One aspect that influences the detection performance comparison of the backgrounds is the dose, or more precisely, the signal and noise magnitude in the images. Although a standard metric that is important for system characterization and comparison, mean glandular dose (MGD) was not used for this comparison for two reasons. First, we are not able to calculate the MGD for the mono‐energetic case as MGD uses conversion factors estimated for broad beam incident spectra. Second, MGD will only enable a meaningful comparison of x‐ray detector signal and noise if the x‐ray spectrum, x‐ray transmission, and glandular compositions are the same. Using x‐ray detector signal and noise directly allows a comparison of these factors and their influence on microcalcification detection.

The mean SI for the L1 phantom is lower than that for the patient group. This may be due to the thickness/attenuation dependence for the Pristina AOP design, which systematically reduces SI for thicker objects. We assume this has happened for the L1 phantom, giving a lower SI for L1 than for the average breast images. This will also have some influence on the image noise and detection performance. For a homogeneous background, the change in threshold contrast detectability would follow the Rose model with power term 0.5,^55^ assuming a quantum noise‐limited system, which is the case here. For the L1 structured background, Vancoillie et al.^22^ found a power term of approximately 0.2, indicating a weaker relationship for threshold contrast detectability as dose changes. Increasing the SI for the L1 phantoms to bring the values closer to those for the SSBT and patient data would give slightly higher detection performance, although giving a value is difficult to estimate.

For the patient backgrounds, a larger range is seen in SI, and this could be due to a number of reasons. First, there is greater variation in tissue types in the breast compared to the phantoms. Therefore, it is likely that transmission averaged over the 40 mm × 40 mm ROI is subject to more variation. The second source of variation may be linked to AOP operation. The Senographe Pristina AOP ensures that the entrance air kerma to the detector reaches a predefined level in the most attenuating region in the field of view. Consequently, the variations in local dense breast regions will increase the variability in SI.

Table 4 shows that there is a corresponding increase in the beta coefficient, from 2.06 to 2.81, as VBD increases, consistent with previous studies.^54^ Our data support earlier observations that higher values of beta are associated with some reduction in the fraction of correctly detected microcalcifications. It must be noted that the beta coefficient for the SSBT background is significantly higher than for the patient backgrounds (3.33 vs. 2.49) and conversely, beta is lower for the physical L1 phantom (2.22 vs. 2.49). The beta coefficient quantifies the spatial correlation of structures in the low frequency range, over the range of 0.08‐0.81 mm^−1^.^38^, ^39^, ^43^ in this study. In a Fourier‐based spatial frequency description, small objects such as microcalcifications will have a relatively large fraction of the signal spectrum at higher spatial frequencies.^56^ This highlights a limitation of the beta coefficient when used as a single parameter to assess the realism of breast‐simulating images, although this metric has been used in many studies involving projection imaging, DBT and reconstructed breast CT images.^9^, ^10^, ^14^, ^27^, ^36^, ^39^, ^43^, ^54^, ^57^, ^58^, ^59^, ^60^ The beta values for patient images in this work are broadly consistent with those in the literature.^36^ This work found some relationship between the fraction of detected microcalcification clusters and beta for the patient images, but the relationship was not consistent for the SSBT and L1 background images. Judged objectively using beta, the phantom images would be rated as having limited realism, however, evaluated using a microcalcification detection task, equivalent or only small differences in observer AUC were found.

The patient backgrounds had the lowest true positive rate and the highest false positive rate, reflecting the greater range of structures present in real breast tissue that can obstruct target visibility. In addition to fibroglandular and adipose material, there is skin, fibrous ligaments, ducts, and blood vessels.^61^ These structures are not present in L1 nor in the SSBT backgrounds, although medium and small‐scale glandular and adipose structures are modeled.^27^ Adding these extra finer structures may increase the magnitude of the power spectrum at higher spatial frequencies and help to bring the beta coefficient closer to that of patients. It should be noted that only 3 out of the 12 available SSBT texture types were used in this study.^27^ Although Cooper's ligaments are not simulated in L1 backgrounds, the intersections of adjacent spheres create finer linear structures and complexity compared to the SSBT phantoms. This might explain the slight increase in false positives for L1 compared to SSBT backgrounds. Detection fractions for the SSBT and L1 phantom most closely agreed with results for the patient backgrounds with homogeneous texture distribution, that is, uniformly distributed breast structures. The lack of anatomical noise related to local areas of glandular tissue and fine structures, which could be seen as a potential calcification cluster, may have simplified the detection task in the phantom backgrounds examined in this work.

Figure 8 shows that there is some influence of VBD on the correctly detected fraction of microcalcification lesions. In a study by Mackenzie et al.,^62^ using a four‐alternative forced choice method, VBD had only a small effect on microcalcification detection. Badano et al.^14^ found in his in‐silico imaging trial no significant difference in AUC for the detection of one specific microcalcification cluster in DBT images across the four density categories. In the present study, the detection fractions for the SSBT and L1 phantoms most closely matched the detection performance in patient backgrounds in regions with the lowest levels of glandular tissue. However, for the SSBT phantom, there is also a reasonably good match with patient background regions of density group 2. We emphasize that a direct comparison should not be made between actual VBD values estimated using Volpara and the calculated glandularity of the SSBT phantoms, as the latter is the exact ratio of the numbers of voxels assigned as glandular and adipose tissue.

In this work, the difference in AUC scores between the L1 and patient backgrounds exceeded the preset margin of 0.1 (*p* = 0.06 for physical L1 and *p* = 0.90 for digital L1). However, the AFROC curve of the physical and digital L1 phantom was close to that of the patients. The use of a detection task as a means of evaluating realism has shown the L1 background to be much closer to real clinical images than judging image appearance/structures alone. Despite its simple design, the phantom enables a quick and direct assessment of DBT imaging performance for a device installed at a particular clinical site. The phantom is easy to fabricate, does not suffer from artifacts, and generates a unique background for each image acquisition by shaking the phantom. Previous work has demonstrated the value of the L1 phantom in routine testing and system comparison.^15^, ^22^ Conversely, a physical version of the SSBT phantom might offer closer realism to patient backgrounds but is difficult to produce, currently lacks a lesion set, and must be handled carefully to avoid introducing artefacts.^63^

Some limitations apply to this study. First, the images were read by medical physicists and not by radiologists. We believe the use of physicists is justified as no clinical knowledge is needed, with the observer only performing a basic localization task over a limited area of 40 mm × 40 mm. The readers had substantial experience in performing similar detection task‐based reading studies and underwent appropriate training prior to the study, in which they were familiarized with the detection task and with the different background types.

A second limitation is that scattered radiation was not modeled in CatSim, and consequently not included in the SSBT and digital L1 backgrounds, or in the microcalcification templates used for all four backgrounds. As a result, the contrasts of the templates will be higher than would be the case physically, however, this applies equally to all backgrounds. It is therefore possible that the detection task will be slightly more difficult in the SSBT and digital L1 backgrounds as there is no scatter signal that would smooth the signal modulation across the images. This is a bias when comparing the detection performance in real and simulated images. The scatter‐to‐primary‐ratio (SPR) is in the range of 0.05–0.12 at this energy and these thicknesses^32^, ^64^ for this DBT system with anti‐scatter grid, and therefore the effect on detection is likely to be small.

Third, the CatSim tool used in this work only employed a mono‐energetic approximation (23 keV) to the exit spectrum of the real system. This is higher than the mean energy of the poly‐energetic input spectrum (approximately 19.7 keV) but close to the energy of the poly‐energetic exit spectrum, which depends on the breast thickness and composition (approximately 22.8 to 23.5 keV, for the thickness range here). The mono‐energetic approximation will increase or decrease the contrast between the glandular and adipose compartments of the SSBT phantom, depending on the local tissue composition. When generating the calcification cluster templates, we did not account for variations in background tissue attenuation, however, this is only a small effect of approximately 2% on lesion contrast in the projections, depending on the background composition.

Furthermore, a mono‐energetic simulation can potentially underestimate the modeled noise. First, there will be an increase in noise due to the Swank effect.^65^ The Swank factor was not explicitly included in the CatSim version used,^32^ however, noise was calibrated based on SNR measurement on a real Pristina system. As a result, additional noise from gain variations in the scintillator is therefore included via this calibration step. Second, mono‐energetic simulations tend to underestimate image noise, with the extent depending on the spectrum width and the imaging task.^66^ This effect will be limited for the relatively narrow Rh/Ag spectrum used in the Pristina system.

Additionally, just two breast phantoms were studied, while there are other digital and physical phantoms available. The SSBT textured phantom is currently generated as a small cuboid volume due to memory constraints; future work will look at techniques to generate phantoms with a more realistic breast size and shape. Finally, one DBT device was implemented, with a specific set of x‐ray technique factors, x‐ray detector, and reconstruction algorithm. Extension to other x‐ray devices with different system parameters would require additional validation studies.

## 5 CONCLUSION

This study has compared human reader detection performance of microcalcification clusters in DBT images of three phantom backgrounds to real patient backgrounds. Only the SSBT phantom had an AUC that was equivalent to the patient images. These results demonstrate the utility of phantoms in optimization work even when absolute detection fractions are required as an endpoint. For the DBT system, reconstruction algorithm, and simulation framework employed in this study, the SSBT phantom has the potential to replace or augment patient data in microcalcification detection studies.

## CONFLICT OF INTEREST STATEMENT

The authors declare no conflicts of interest.

## Supporting information



Supporting Information

Supporting Information
