# Supplementary figures and images for: A virtual imaging study of microcalcification detection performance in digital breast tomosynthesis: Patients versus 3D textured phantoms
Source: Med Phys. 2025 May 8;52(6):3800–14. doi: 10.1002/mp.17873 (PMC12149725; doi:10.1002/mp.17873)

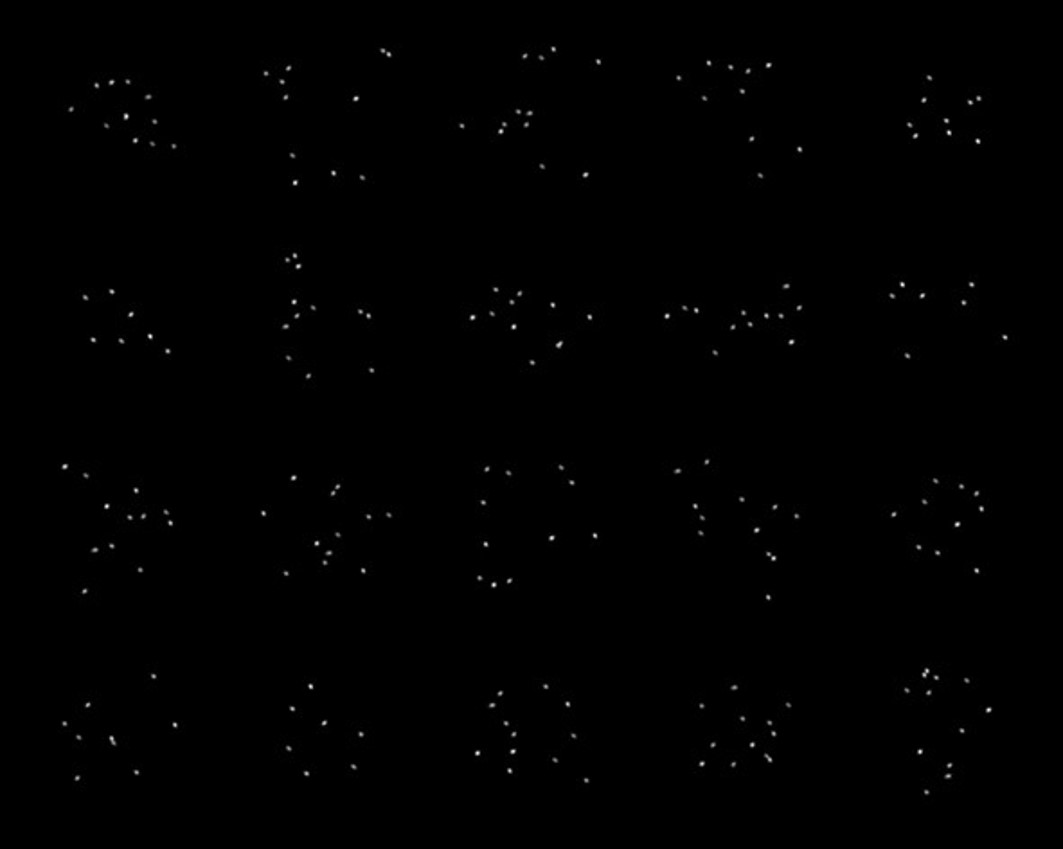

Supplement: Supplementary file 1 — Supporting Information [file MP-52-3800-s001.jpg]

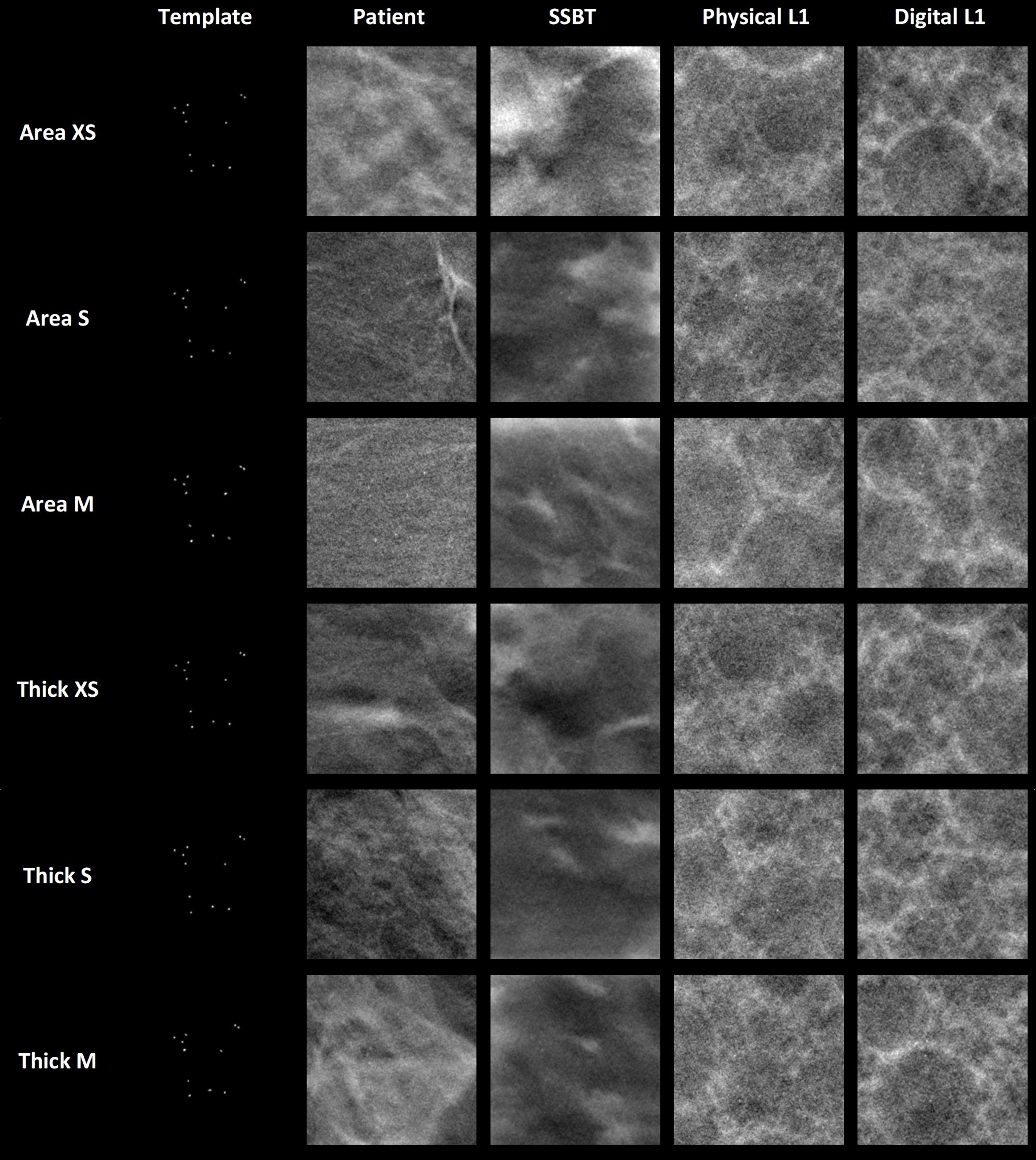

Supplement: Supplementary file 2 — Supporting Information [file MP-52-3800-s002.jpg]
